# The Functions of BET Proteins in Gene Transcription of Biology and Diseases

**DOI:** 10.3389/fmolb.2021.728777

**Published:** 2021-09-03

**Authors:** Ka Lung Cheung, Claudia Kim, Ming-Ming Zhou

**Affiliations:** Department of Pharmacological Sciences, Icahn School of Medicine at Mount Sinai, New York, NY, United States

**Keywords:** bromodomain, bromodomain and extra-terminal domain proteins, gene transcription, inhibitors, cancer

## Abstract

The BET (bromodomain and extra-terminal domain) family proteins, consisting of BRD2, BRD3, BRD4, and testis-specific BRDT, are widely acknowledged as major transcriptional regulators in biology. They are characterized by two tandem bromodomains (BDs) that bind to lysine-acetylated histones and transcription factors, recruit transcription factors and coactivators to target gene sites, and activate RNA polymerase II machinery for transcriptional elongation. Pharmacological inhibition of BET proteins with BD inhibitors has been shown as a promising therapeutic strategy for the treatment of many human diseases including cancer and inflammatory disorders. The recent advances in bromodomain protein biology have further uncovered the complex and versatile functions of BET proteins in the regulation of gene expression in chromatin. In this review article, we highlight our current understanding of BET proteins’ functions in mediating protein–protein interactions required for chromatin-templated gene transcription and splicing, chromatin remodeling, DNA replication, and DNA damage repair. We further discuss context-dependent activator *vs.* repressor functions of individual BET proteins, isoforms, and bromodomains that may be harnessed for future development of BET bromodomain inhibitors as emerging epigenetic therapies for cancer and inflammatory disorders.

## Introduction

**FIGURE 1:**
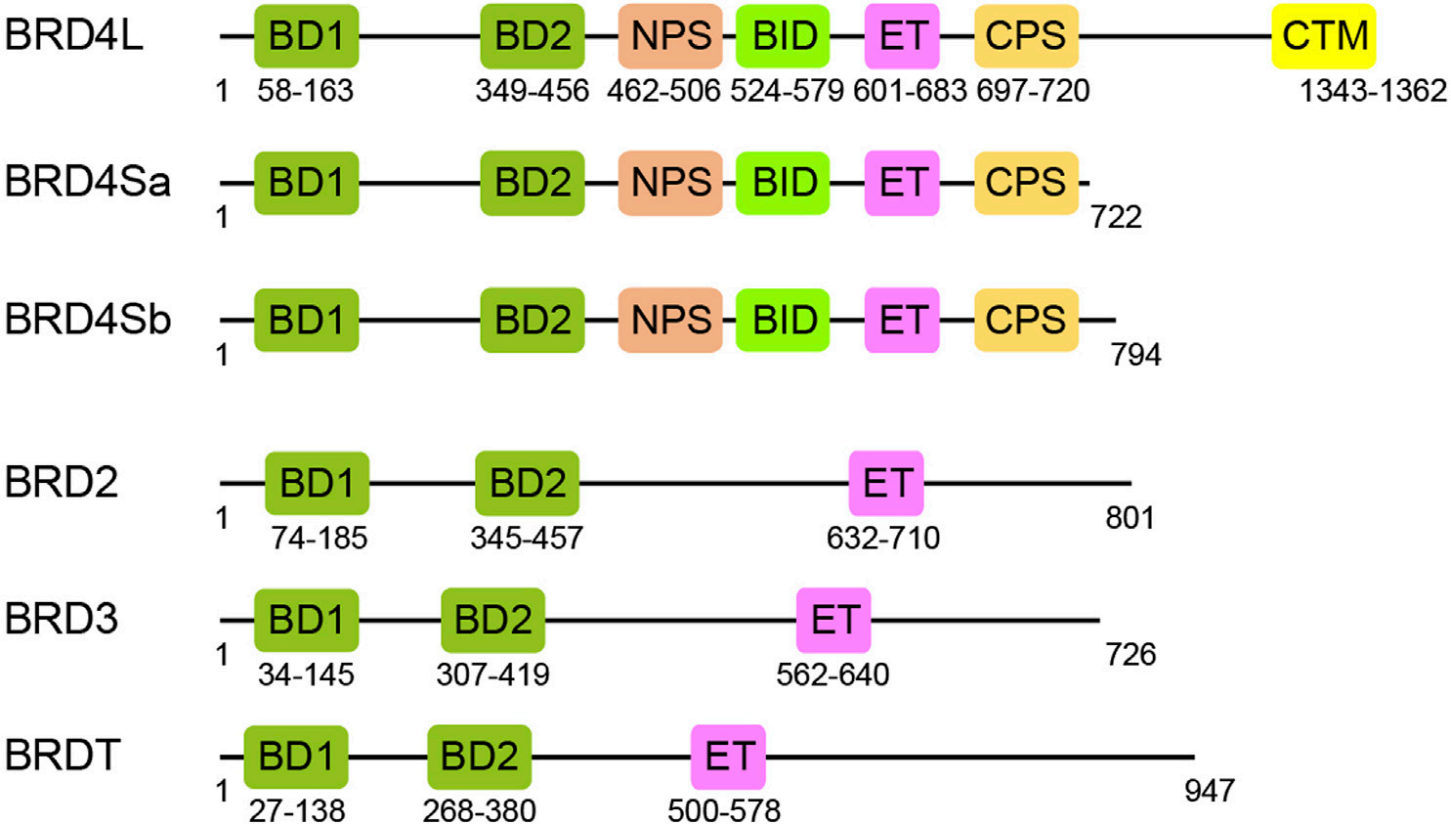
Functional domains of BET family proteins. Schematic representation of domain architectures for BET proteins, BRD4 long and short isoforms, BRD2, BRD3, and BRDT: BD1, first bromodomain; BD2, second bromodomain; NPS, N-terminal phosphorylation site; BID, basic residue-enriched interaction domain; ET, extra-terminal; CPS, C-terminal phosphorylation site; CTM, C-terminal motif.

Bromodomain and extra-terminal domain (BET) family proteins, consisting of BRD2, BRD3, BRD4, and BRDT, are important transcription regulators, characterized by two N-terminal acetyl-lysine (Kac) binding bromodomains (BD1 and BD2) followed by an ET domain (Wu and Chiang, 2007; Zaware and Zhou, 2019) (Figure 1). BRD4 exists in three isoforms BRD4L, BRD4Sa, and BRD4Sb that reportedly have distinct functions in gene transcription (Wu et al., 2020). The C-terminal motif (CTM) is unique in BRD4L and not present in BRD4S and other BET proteins. Besides BD1, BD2, and CTM, BET proteins also have several functional motifs including a cluster of casein kinase 2 (CK2) phosphorylation sites (NPS and CPS) and a basic residue-enriched interaction domain (BID) (ChiangPhospho-BRD4, 2016). As arguably the most well-characterized domains in BET proteins, BD1 and BD2 domains, composed of the evolutionarily conserved four-helical bundle structure, function to recognize acetylated lysine in histones or transcription factors and act as readers of acetylation signals in gene transcription in chromatin (Dhalluin et al., 1999). The ET domain confers transcriptional activation by recruiting transcription regulatory proteins (Rahman et al., 2011; Zhang et al., 2016). Although the BET family proteins share similar functional domains (BD1, BD2, and ET), their expression patterns in tissues are distinct (Wang et al., 2021a). BRD2 and BRD3 are found to be expressed in the pancreas, testis, ovaries, brain, liver, spleen, lung, and kidney (Shang et al., 2009; Wang et al., 2009; Wang et al., 2021a). BRD4 is ubiquitously expressed, as it is functionally expressed in the bone marrow and lymphoid compartment in addition to other tissues, while BRDT is selectively expressed in the testis and ovaries (Wang et al., 2021a). In cells, BET proteins also display non-redundant functions, which are often cooperative in activation of gene transcription (Cheung et al., 2017a). Therefore, it is important that we gain a deeper understanding of the overlapping and distinct functions of BET proteins in gene transcription and diseases.

The most well-studied function of BET proteins, particularly BRD4, is the role in transcriptional elongation (Hargreaves et al., 2009). In a simplistic model of transcriptional activation, RNA polymerase (Pol)-II recruitment to regulatory sites such as enhancers and promoters is the first limiting step to activate gene transcription. This simple model has been challenged and refined by the concept of transcriptional elongation, which accounts for the fast kinetics of gene activation and transcriptional memory upon stimuli. In a seminal study by Medzhitov and colleagues using bone marrow–derived macrophages to address the difference in transcriptional kinetics of primary response genes (PRGs) and secondary response genes (SRGs), it was found that PRGs, but not SRGs, have pre-assembled RNA Pol-II at their promoters in unstimulated conditions. Upon stimulation, signal-dependent recruitment of BRD4/p-TEFb to acetylated histones of these sites facilitates RNA Pol-II transcriptional elongation and mRNA processing (Hargreaves et al., 2009). In addition to direct effect on promoter sites, BRD4 also exerts pronounced effect on anti-pause enhancers (A-PEs) that activates promoter-bound RNA Pol-II through a mechanism termed “transcriptional pause-release” (Anand et al., 2013; Liu et al., 2013). BRD4 recruits demethylase JMJD6 on A-PEs to erase H4R3me2s, which is read by 7SK snRNA, and demethylation/deactivation of 7SK small nuclear RNA (snRNA), to inhibit the binding and function of the 7SK snRNA/HEXIM repressive complex, therefore allowing for pause-release of RNA Pol-II (Liu et al., 2013). In addition to their role in gene transcriptional elongation, recent technological advancements have also led to the discovery of other functions of BET proteins, including chromatin organization, super-enhancer assembly, and condensate formation. In this review article, we first discuss in detail various molecular mechanisms that modulate BET protein expression and activity in human diseases, which are not well-appreciated and lack systematic review. We then summarize the recent advances in our understanding of the multi-faceted functions of BET proteins in gene transcription in chromatin. Finally, we highlight the more context-dependent distinct functions of BET proteins including different isoform and repressor functions, providing rationales and opportunities for more selective and potentially less toxic therapeutic strategies targeting BET proteins.

## Molecular Mechanisms Modulating Bromodomain and Extra-Terminal Domain Protein Expression and Function

### Mechanisms Regulating Expression of Bromodomain and Extra-Terminal Domain Proteins

**FIGURE 2:**
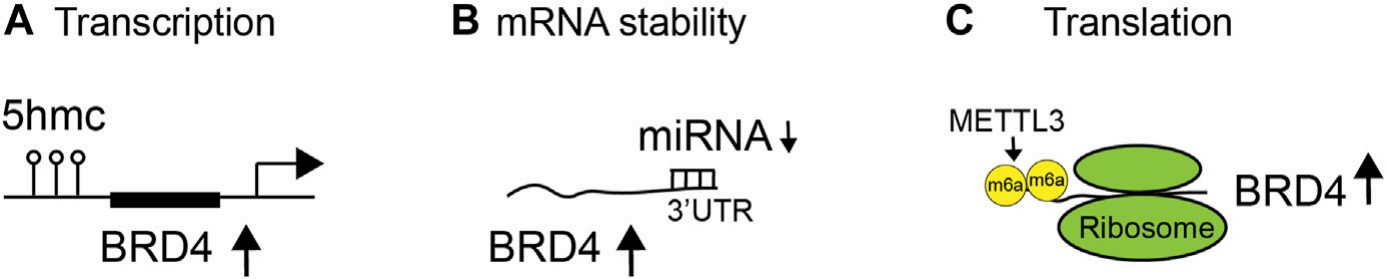
Regulation of BET protein expression and functions in diseases. Expression and activity of BET proteins are regulated at different levels, including **(A)** transcription by 5-hydroxymethylcytosine (5-hmC) at the BRD4 promoter, **(B)** messenger RNA (mRNA) stability by binding of microRNA (miRNA) to the 3′ untranslated region (UTR) of BRD4 mRNA transcripts, and **(C)** translation by *N* ^6^-methyladenosine (m^6^A) modification of mRNA (Rahman et al., 2011).

In cancer, the expression of BET proteins is often upregulated to enhance oncogenic activities. Studies are beginning to reveal exactly how the BET proteins are aberrantly overexpressed in disease contexts. For instance, it has been demonstrated that 5-hydroxymethylcytosine (5-hmC) can regulate BRD4 transcript expression through 5-hmC enrichment at open chromatin and BRD4 promoter sites, correlating with upregulated transcriptional expression of BRD4 in pancreatic neoplasia (Bhattacharyya et al., 2017) (Figure 2A). Additionally, miRNA can bind to the 3′ untranslated region (UTR) of BRD4 to regulate the mRNA abundance or stability of BRD4 in diseases (Figure 2B). miR-29b targets the 3′-UTR of BRD4 to regulate the expression of BRD4 and has an inverse correlation (low miR-29b and high BRD4) in chronic obstructive pulmonary disease (COPD) patients (Tang et al., 2019). miR-1340 targets BRD3 and BRD4 to suppress tumor cell growth (Tonouchi et al., 2018). Diminished miR-29b level leads to enhanced BRD4 level and activation in cutaneous T-cell lymphoma (Kohnken et al., 2018). miR-9 suppresses BRD4 expression in cardiomyocytes. In response to pathological stress stimuli, miR-9 is downregulated, leading to de-repression of BRD4 and enrichment of BRD4 on super-enhancers (SEs) associated with pathological cardiac genes (Stratton et al., 2016). Notably, long non-coding RNA (lncRNA) LINC00346, in contrast, acts as a sponge for miR-188-3p and blocks the repression of BRD4 by miR-188-3p in pancreatic cancer (Shi et al., 2019). Finally, upon knockdown of METTL3, endogenous BRD4 expression is strongly reduced, independently of mRNA abundance, leading to the discovery that METTL3-eIF3h mediates *N* ^6^-methyladenosine (m^6^A) modification (Rahman et al., 2011) and circularization of BRD4 mRNA to enhance translation and promote oncogenesis (Choe et al., 2018) (Figure 2C). Collectively, these studies illustrate that BRD4 overexpression in cancer is likely due to enhanced mRNA transcription, abundance, and/or translation.

## Modulation of Bromodomain and Extra-Terminal Domain Protein Functions by Post-Translational Modifications

**FIGURE 3:**
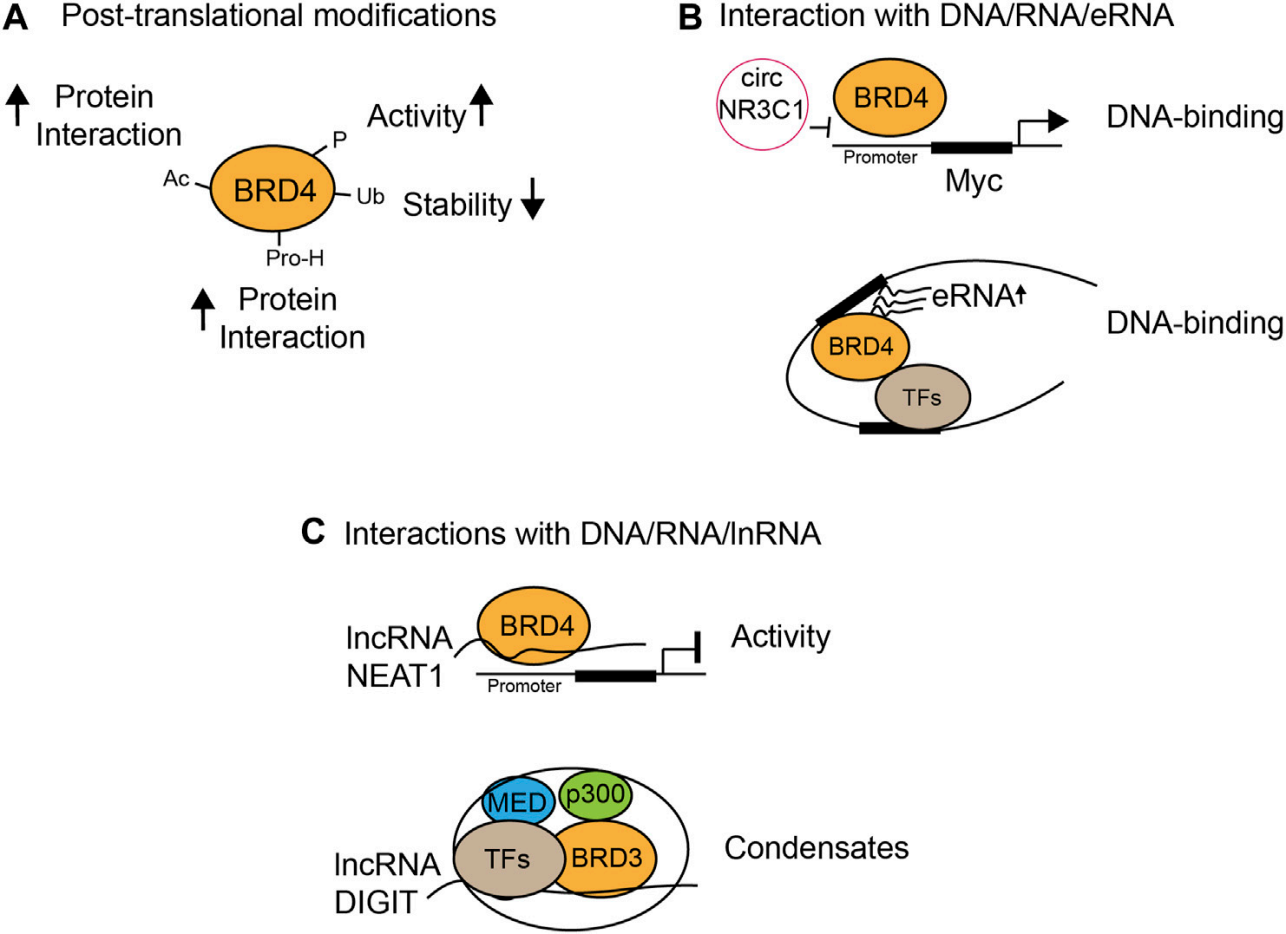
Functional modulation of BET proteins by chemical modifications and protein or nucleic acid interactions. **(A)** Protein stability and activity by post-translational modifications such as acetylation (Ac), phosphorylation (P), ubiquitination (Ub), and proline-hydroxylation (Pro-H); **(B)** modulation of DNA binding through interactions with circular RNA (circRNA) and enhancer RNA (eRNA); **(C)** BRD4 transcriptional activity and condensate formation through interactions with long non-coding RNA (lncRNA).

Post-translational modifications of BET proteins, including acetylation, phosphorylation, ubiquitination, and proline hydroxylation, regulate protein stability and functions (Figure 3A). Acetylation of BRD4 enhances its interactions with other proteins. PCAF acetylates transcription factor ISX at Lys69 and BRD4 at Lys332, which form a complex and translocate into the nucleus to promote transcriptional expression of genes important for epithelial–mesenchymal transition and metastasis (Wang et al., 2020). Phosphorylation of BRD4 (pBRD4) is often associated with enhanced BRD4 oncogenic activities in cancer, as demonstrated by hyper-phosphorylated BRD4 in NUT midline carcinoma, potentially mediated by CDK9 (Wang et al., 2017). In addition, kinase (CK2) and phosphatase (PP2A) that phosphorylate and dephosphorylate BRD4, respectively, to modulate its function in chromatin localization, transcription factor recruitment, and cancer progression have been reported (ChiangPhospho-BRD4, 2016). Importantly, CK2-mediated phosphorylation of a conserved acidic region in BRD4 dictates whether this region contacts with juxtaposed bromodomain (BD2) or an adjacent basic region, therefore determining if BD2 is free to bind acetylated-TF p53 (Wu et al., 2013). The same mechanism also applies in cocaine-seeking behavior and memory function in mice, as cocaine or neuronal stimulation induces the activation and binding of pBRD4 to the key gene promoter, which can be attenuated by CK2 blockade (Korb et al., 2015; Guo, 2020). Therefore, apart from modulating BRD4 bromodomains’ interactions with chromatin, strategies targeting activated pBRD4 may represent an alternative and promising approach.

Equally important as acetylation and phosphorylation is BRD4 ubiquitination that has been linked to aberrant degradation of BRD4 leading to BET-BD inhibitor resistance. The most well-characterized example of BRD4 ubiquitination is controlled by the SPOP-DUB3 switch. De-ubiquitinase DUB3 binds to BRD4, but not BRD2/3, via a specific C-terminal motif (CTM), antagonizes SPOP-mediated BRD4 ubiquitination, and promotes BRD4 de-ubiquitination and stabilization, leading to BET-BD inhibitor resistance in cancer cells (Jin et al., 2018). In prostate cancer, specific SPOP mutation can also confer resistance to BET-BD inhibitors through stabilization of BRD4 (Dai et al., 2017) and enhanced Akt–mTORC1 activation as a result of BRD4 stabilization (Zhang et al., 2017). Interestingly, SPOP mutants in different types of cancer yield opposing effects on BET protein degradation by differential ubiquitination and sensitivity to BET-BD inhibitors. Endometrial cancer–associated SPOP mutations preferentially degrade BET proteins, while prostate-cancer–specific SPOP mutation results in impaired degradation of BET proteins, as mutations of SPOP degrons in BRD4 determine whether they bind BET proteins or not, thus influencing BET ubiquitination (Janouskova et al., 2017). Finally, the less well-characterized post-translational modifications of BRD4 include proline hydroxylation and isomerization. Prolyl hydroxylase domain protein PHD2 is the key regulatory enzyme of BRD4 proline hydroxylation (P536) that significantly influences BRD4 interaction with CDK9, CDK1, and MCM5 and affects BRD4 transcriptional activation, but not BRD4 degradation or abundance (Erber et al., 2019). Prolyl isomerase PIN1 regulates the stability and transcriptional activity and oncogenic potential of BRD4 in gastric cancer cells. PIN1 recognizes BRD4 Thr204 and enhances BRD4 stability by inhibition of ubiquitination. PIN1 also catalyzes the isomerization of BRD4 Pro205 and induces its conformation change to promote CDK9 interaction and transcriptional activity and gastric cancer cell proliferation (Hu et al., 2017).

## RNA/DNA-Mediated Mechanisms Modulating Functions of Bromodomain and Extra-Terminal Domain Proteins

It has been increasingly recognized that interactions of BET proteins with RNA/DNA determine the functional outcomes of BET proteins in gene transcription. For example, RNA has been shown to regulate the DNA-binding activity or transcriptional activity of BRD4 in different contexts. Circular RNA circNR3C1 dissociates BRD4 from binding to the c-Myc promoter in bladder cancer cell lines and suppresses bladder cancer progression as an endogenous blocker of BRD4 (Xie et al., 2020) (Figure 3B). On the contrary, lncRNA could inhibit BRD4 transcriptional activity. BRD4 binds several long non-coding RNAs, and one of them is lncRNA NEAT1 that interacts with BRD4/WDR5 and forms a low-activity complex. BET-BD inhibitors dissociate NEAT1 from BRD4/WDR5 and restore the acetyltransferase activity of BRD4 and promote WDR5-dependent histone trimethylation, leading to transcriptional activation (Pistoni, 2021) (Figure 3C). Interestingly, BRD3 forms phase-separated condensates, of which formation is promoted by lncRNA DIGIT. BRD3–DIGIT co-occupies enhancer sites with histone H3K18ac to cooperatively form protein–lncRNA phase-separated condensates and has a broad role in gene transcription (Daneshvar et al., 2020). Notably, enhancer RNA (eRNA) is another type of RNA that interacts with BET family proteins to promote enhanced chromatin engagement and transcriptional activation. While all BET proteins interact with eRNA through their bromodomains, BRD2, BRD3, and BRD4 interaction with eRNAs is much stronger as compared to BRDT. Specifically, BRD4-BDs function cooperatively as a docking site for direct binding of eRNA to increase BRD4 binding to acetylated histones *in vitro*, BRD4 enhancer recruitment, and transcriptional coactivator activities (Rahnamoun et al., 2018). eRNA production is a key feature of enhancer activation and is BRD4-dependent as the BET-BD inhibitor JQ1 reduces eRNA abundance on the enhancer, suggesting BRD4 controls a feedback loop that enhances eRNA production on the enhancer, which in turn promotes BRD4 chromatin engagement and transcriptional activation. Finally, it has been shown that interactions of BET-BDs with DNA facilitate acetylation-dependent bivalent nucleosome recognition by BRDT (Miller et al., 2016). BRDT interacts with nucleosomes through its BD1 but not BD2, and acetyl-histone recognition is complemented by a bromodomain–DNA interaction, as simultaneous recognition enhances nucleosome binding affinity and specificity. Conservation of DNA binding in BDs of BET therefore indicates the bivalent nucleosome recognition as a key feature (Miller et al., 2016). Collectively, these results highlight the emerging concept that RNA/DNA-mediated mechanisms play a crucial role in the control of BRD4 chromatin binding and activity that contributes to gene transcriptional controls in diseases.

## Multi-Faceted Functions of Bromodomain and Extra-Terminal Domain Proteins in Gene Transcription in Chromatin

### Histone/Transcription Factor Recruitment of Bromodomain and Extra-Terminal Domain Proteins Through the Bromodomains

**FIGURE 4:**
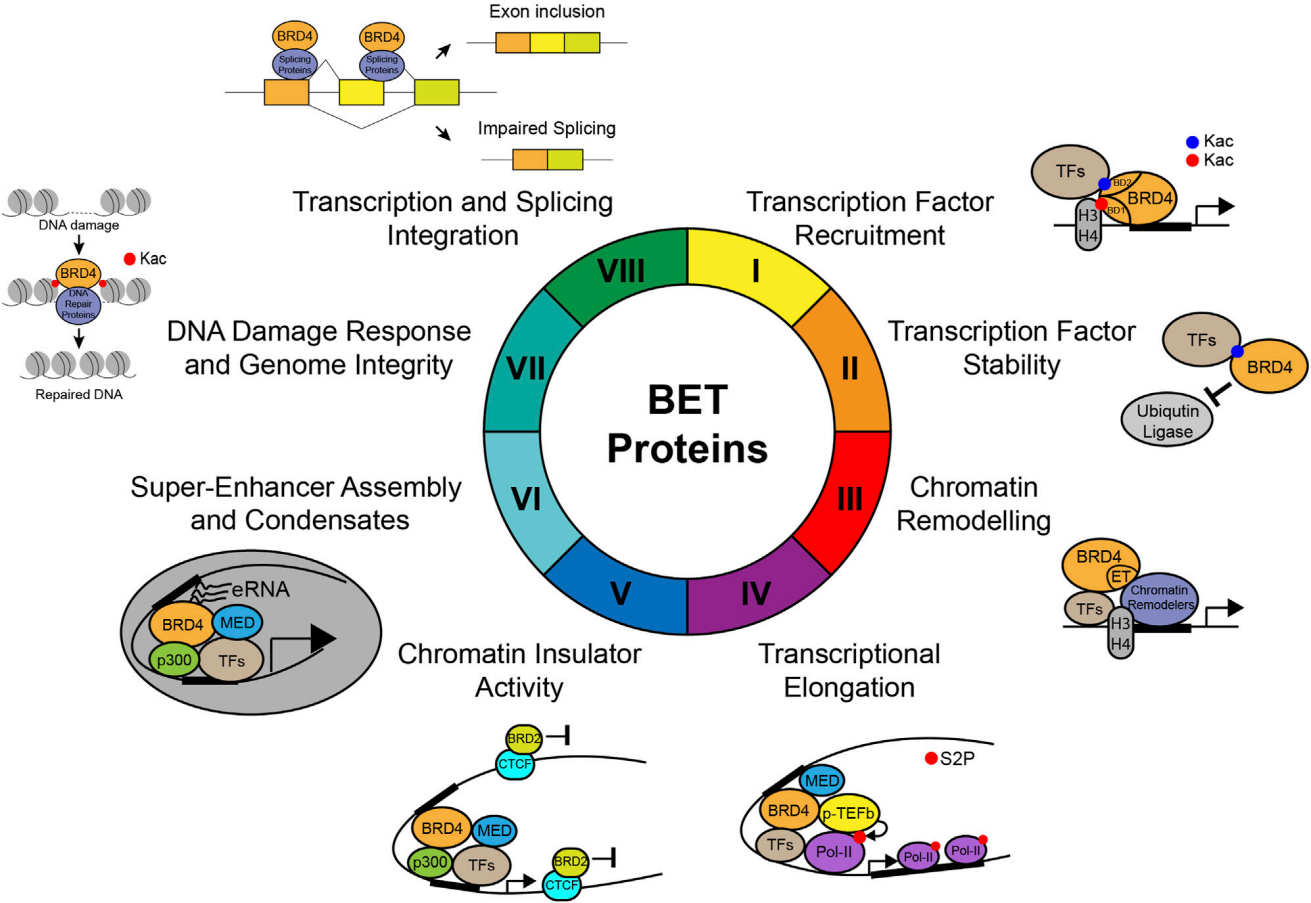
Molecular functions of BET proteins in gene transcription in chromatin. Illustration of the molecular functions of BET proteins in regulation of gene transcription in chromatin, including (I) transcription factor recruitment, (II) transcription factor stability, (III) chromatin remodeling, (IV) transcriptional elongation through phosphorylation of RNA Pol-II Ser2, (V) chromatin insulator activity, (VI) super-enhancer assembly and condensates, (VII) DNA damage response and genome integrity, and (VIII) integration of gene transcription and splicing.

One of the major molecular functions of BET proteins is to act as an epigenetic reader to relay signals from lysine-acetylated transcriptional factors or histones to regulate gene transcription in chromatin (Figure 4). Intriguingly, recent studies also suggested that histone lysine acyl–related modifications such as crotonylation and butyrylation may play a role in BD-mediated recognition (Soffers et al., 2016). While a subset of bromodomain-containing proteins such as BRD9 and TAF1 recognize histone crotonylation and butyrylation, their binding affinities with crotonylated/butyrylated peptides are much weaker than those with acetylated peptides (Flynn et al., 2015). On the contrary, the YEATS domain of AF9 has been shown to strongly bind histone crotonylation compared to acetylation (Li et al., 2016). The BET-BD recognition of acetylation marks instead of other acylation marks is further strengthened by the discovery that histone butyrylation competes to prevent, instead of facilitating, BRDT binding to H4K5 (Goudarzi et al., 2016). Therefore, these studies argue that BDs of BET proteins likely function as a specific reader for lysine acetylation, and not other types of lysine acylation.

Studies have identified different mechanisms as to how BET proteins are recruited to regulatory sites of key genes. Acetylated transcription factors (TFs) and histones bind, recruit, and work with BRD4 to drive super-enhancer formation and gene expression. In acute myeloid leukemia (AML), acetylated histone H4 and hematopoietic transcription factors interact with BRD4 via a common K^ac^GGK^ac^ motif to recruit BRD4 to regulatory sites (Roe et al., 2015). Similarly, the second bromodomain (BD2) of BRD4 interacts with diacetylated Twist via the same motif to promote tumorigenesis in basal-like breast cancer (Shi et al., 2014). The functional transcription factor–BET binding is characterized by conserved binding mechanisms, as each BET-BD1/2 can recognize, in a single binding pocket, a dual-Kac motif of K^ac^XXK^ac^ present in transcriptional factors including TWIST, GATA1, E2F1, and MyoD1 (Patel et al., 2021a). Crystal structures of BD1 of BRDT and BRD2 bound to a diacetylated histone H4 peptide show that two acetylation marks are cooperatively recognized by one binding pocket, and this mode of ligand recognition applies to other bromodomain-containing proteins as well (Nakamura et al., 2007; Morinière et al., 2009). Histone variants are another important regulatory control of aberrant gene transcription in human diseases including cancer. H2A.Z, one of the most well-studied variants, was shown to be co-purified with BRD2. The NMR study has revealed that lysine acetylation on the N-terminal tail of H2A.Z (K4acK7ac and K7acK11ac) interacts with BDs of BRD2, BRD3, and BRD4 and is reminiscent of the characteristic binding of the N-terminal tail of H4-K5acK8ac to BRD4-BD1. Thus, histone variant–BET protein binding also follows the common feature that a single bromodomain preferentially binds diacetylated motifs commonly in a K^ac^XXK^ac^ motif (Patel et al., 2021b). Finally, a systematic proteomics study confirmed that BET-BDs recognize K^ac^XXK^ac^ motifs on histones where XX can be GG, GS, DG, AA, AP, AV, AQ, AR, SA, VL, LN, TA, and TP (Lambert et al., 2019). Interestingly, “histone-like” XX sequences were found in the human proteome, recognized by BD1 and/or BD2 domains, and a diverse flanking sequence can drive binding to either bromodomain (Lambert et al., 2019). These studies highlighted the binding specificity and versatility of BET-BD1/2 domains and K^ac^GGK^ac^ motif in both histones and non-histone proteins.

Apart from the simple paradigm that H4K5/8/12 acetylation mediates BRD4 binding, more complex histone crosstalk and combinatorial histone modifications create a platform for BRD4 binding. For example, serum stimulation of cells induced PIM1 kinase to phosphorylate the pre-acetylated histone H3 (H3K9ac/S10ph) at the FOSL1 enhancer. 14-3-3 then acts as an adaptor protein that binds the phosphorylated histone to further recruit the histone acetyltransferase MOF and trigger the acetylation of H4K16ac for BRD4 binding (Zippo et al., 2009). By screening over 30 representative BD proteins against histone peptide arrays with systematic changes in histone combination marks, it was confirmed that BRD4 recognizes a combination of marks such as acetylation and phosphorylation rather than singly acetylated sequences (Filippakopoulos et al., 2012). Thus, BRD4 binding can be a result of a cascade of events leading to a nucleosomal platform that recruits BRD4.

## Bromodomain and Extra-Terminal Domain Proteins Regulate Stability of Transcription Factors

BRD4 interaction with transcription factors can enhance binding partner stability or induce degradation through different mechanisms (Figure 4). In castration-resistant prostate cancer (CPRC), the histone demethylase JMJD1A was shown to regulate activities of androgen receptor (AR) and c-Myc. JMJD1A protein stability is controlled by ubiquitin ligase STUB1. Histone acetyltransferase (HAT) p300 acetylates JMJD1A at K421 that recruits BRD4 to block JMJD1A from degradation, which is reversed by p300 HAT or BET-BD inhibitors (Xu et al., 2020). Similarly, BRD4 C-terminal domain (CTD) interacts with HPV16 (human papillomavirus) E2 protein, which blocks E2 interaction with cullin-3, leading to reduced E2 ubiquitination and enhanced E2 stability (Zheng et al., 2009). Finally, BRD4 recognizes K146ac and K187ac on Snail to prevent Snail from interacting with E3 ubiquitin ligase FBX14 and b-Trcp1, thereby inhibiting Snail polyubiquitination and proteasomal degradation, revealing a non-canonical post-transcriptional regulatory function of BRD4 in maintaining cancer cell growth and metastasis (Qin et al., 2019). Conversely, BRD4 was also shown to induce degradation by phosphorylation of TFs. It has been reported that oncogenic protein MYC stability is controlled by phosphorylation, as pThr58 signals degradation, while pSer62 leads to stabilization. BRD4, with its reportedly intrinsic kinase and histone acetyltransferase activity, phosphorylates MYC at Thr58, leading to MYC ubiquitination and degradation (Devaiah et al., 2020). These studies suggest one major mechanism of BET proteins in acetylation-dependent stabilization of interacting TFs, which likely competes off the binding of E3 ubiquitin ligases.

## Bromodomain and Extra-Terminal Domain Protein Interactions With Chromatin Remodelers Through the ET Domain

The ET domain of BET proteins has been shown to be involved in protein–protein interactions in gene transcription (Figure 4). As shown by a proteomic analysis, the ET domain of BRD4 interacts with multiple chromatin remodeling proteins including NSD3, JMJD6, CHD4, and GLTSCR1 (Rahman et al., 2011). As demonstrated by further detailed structural and functional characterization (Liu et al., 2013; French et al., 2014; Shen et al., 2015; Zhang et al., 2016; Konuma, 2017; Gatchalian, 2018; Wong, 2019; Lam, 2020; Lee, 2020), the ET domain recognizes a short peptide motif that is conserved across chromatin remodelers and transcriptional regulators. This is supported by a study showing that BRD3 binds to an array of chromatin remodeling complexes, including the NuRD, BAF, and INO80 complexes, via a short linear “KIKL” motif in one of the complex subunits (Wai et al., 2018). In addition, the ET domain–binding motif, which shares similar amino acid sequences from other BRD4-ET–binding proteins such as CHD4 and NSD2/3, also mediates interaction of BRD4-ET with ASXL3 and functionally links BRD4 to the BAP1 complex in a small-cell lung cancer (SCLC) subtype (Szczepanski, 2020). Interestingly, the detailed NMR structural analysis of JMJD6/BRD4-ET domain complex by Zhou and colleagues revealed that a JMJD6 peptide (Lys84–Asn96) adapts an α-helix when bound to the conserved hydrophobic core of the ET domain (Konuma, 2017). This ligand recognition mode is different from the ET domain recognition of NSD3, LANA of herpesvirus, and integrase of MLV that proceeds with the formation of a small two-strand β-sheet (Zhang et al., 2016; Konuma, 2017). Collectively, these studies provided the structural and molecular basis on how the BRD4-ET domain recognizes a conserved BRD4-binding motif on chromatin remodeling complex proteins to form a functional complex for gene transcription.

## Bromodomain and Extra-Terminal Domain Proteins Facilitate RNA Pol-II Pause-Release and Transcriptional Elongation

BET proteins are known to facilitate RNA Pol-II release from promoter-proximal pause regions through recruitment and activation of p-TEFb (CDK9/cyclin T1) that triggers transcriptional elongation (Yang et al., 2005; Jang et al., 2005) (Figure 4). BRD4 association with p-TEFb is required to form an active complex for stimulation of RNA Pol-II elongation on both coding and non-coding enhancer RNAs (Kanno et al., 2014), and the functional importance has been demonstrated in different cellular contexts including embryonic stem cells (ESCs) (Di Micco et al., 2014), heart cells (Anand et al., 2013), and macrophages (Hargreaves et al., 2009). Mechanistically, BRD4-BD2 interactions with tri-acetylated cyclin T1 (K380ac, K386ac, K390ac) and cyclin T1 with BRD4-PID (C-terminal p-TEFb–interacting domain) are required for the release of p-TEFb from an inactive HEXIM1-7SK small nuclear ribonucleoprotein complex and for full transcriptional activation of p-TEFb (Schröder et al., 2012). In addition to BRD4, p-TEFb can also be recruited to target genes by a super-elongation complex (SEC) in the development of cardiomyocyte hypertrophy following activation of distinct GPCR [α_1_-adrenergic receptor (α_1_-AR) or endothelial receptor (ETR)]. However, while the SEC is required for both activation modes, BRD4 is selectively required for α_1_-AR response, suggesting the BRD4/p-TEFb complex can be differentially activated in response to distinct signaling pathways (Martin, 2020).

Importantly, BRD4 is involved in multiple steps of the transcriptional elongation hierarchy. Using an auxin-inducible degradation (AID) system to rapidly deplete targeted proteins, it was shown that CDK9-containing BRD4 is required for genome-wide release of RNA Pol-II from promoter-proximal pausing under normal conditions, while BRD2 is required for global RNA Pol-II positioning at enhancers (Zheng et al., 2021). As aforementioned, in addition to coding genes, BRD4 mediates its function through enhance regions, as it assists enhancer RNA synthesis through transcriptional elongation. In addition, BRD4 was also shown to interact with JMJD6 (a histone arginine demethylase) to demethylate H4R3 at the target gene anti-pause enhancer to release RNA Pol-II from promoter-proximal pause regions, leading to aberrant gene expression in glioblastoma (Wong, 2019). However, although BET and mediator proteins have been shown to form phase condensates and are crucial for super-enhancer function, dissolving phase condensates reduces BRD4 and mediator binding to enhancers but does not disrupt enhancer–promoter interactions (Crump, 2021). In addition, targeting cells with BET-BD inhibitors has a strong impact on transcription but very little impact on enhancer–promoter interactions (Crump, 2021). These findings suggest that activation of transcription and maintenance of enhancer–promoter interactions are separable events and that BRD4 acts in activation of gene transcription through p-TEFb–mediated RNA Pol-II release from enhancer and promoter regions much more than in the maintenance of enhancer–promoter interactions.

## Chromatin Structure Organization by Bromodomain and Extra-Terminal Domain Proteins

BET proteins actively organize chromatin to define transcriptional and architectural boundaries for gene transcription (Figure 4). Chromatin architectural/insulator protein CTCF recruits BRD2, but not BRD4, and they colocalize genome-wide to insulate regulatory influence from unrelated genes, indicating that CTCF and BRD2 form a transcriptional boundary, of which activity is dependent on BRD2 (Hsu et al., 2017). This is further supported by preferential binding of BRD2 to CTCF/cohesin components, illustrating the distinct functions between BRD2 and BRD4 in that BRD2 are involved in boundary activities, while BRD4 promotes transcriptional elongation (Cheung et al., 2017a). Although BRD2’s function in chromatin organization is evident, BRD4’s ability to associate with acetylated chromatin is nonetheless also important for maintenance of higher order chromatin structures and defining transcriptional boundaries (Wang et al., 2012). It has been reported that BRD4 interacts and forms a complex with p300/CBP through its BDs to enhance its C-terminal intrinsic HAT activity, which enhances p300 enzymatic activity to deposit H3K27 and H3K56 acetylation, regulates Brg1 binding to chromatin, and thus modifies the chromatin structure (Wu et al., 2018). In NUT (nuclear protein in testis) midline carcinoma (NMC), a highly aggressive subtype of squamous cell cancer, BRD4–NUT fusion oncoprotein enhances the interaction of p300 and BRD4, leading to a massive feed-forward expansion of transcriptionally inactive hyperacetylated chromatin domains (Reynoird et al., 2010). Finally, BET proteins also actively evict histones to facilitate gene transcription. For example, interferon (IFN) stimulation induces GCN5-mediated acetylation of H2A.Z, leading to BRD2 engagement, chromatin remodeling, and eviction of H2A.Z. This in turn enables ISGF3 to bind and activate interferon-stimulated gene (ISG) expression, providing an example of how BRD2 can evict nucleosomes to facilitate gene transcription (Au-Yeung and HorvathHistone, 2018). BRD4 has reported intrinsic HAT activity that acetylates histone H3K122, a residue critical for nucleosome stability, and evicts nucleosomes from chromatin, resulting in chromatin decompaction, thus linking the chromatin structure and gene transcription (Devaiah et al., 2016). Similarly, PHD finger protein 7 (PHF7) mediates BRDT stability (Kim et al., 2020), which is required for recognition of acetylated chromatin through the BD1 of BRDT (Dhar et al., 2012) and removal of histones to facilitate histone-to-protamine exchange, a critical step for sperm nuclear condensation during spermatogenesis (Kim et al., 2020). These studies together demonstrate the critical histone removal functions of BET proteins in different functional contexts.

## Bromodomain and Extra-Terminal Domain Proteins in Super-Enhancer Assembly

Pharmacological inhibition of the BET bromodomains has been regarded as a new therapeutic strategy targeting the transcriptional expression of c-Myc–dependent and Myc-dependent target genes. JQ1 is among the best known bromodomain inhibitors that effectively displaces BRD4 interaction from acetylated histones (Filippakopoulos et al., 2010). *In vivo* efficacy of JQ1 in cancer cell growth inhibition has been confirmed in mouse models of various cancers including squamous carcinoma (Filippakopoulos et al., 2010), multiple myeloma (Delmore et al., 2011), acute myeloid leukemia (AML) (Zuber et al., 2011), MLL-fusion leukemia (Dawson et al., 2011), castration-resistant prostate cancer (Asangani et al., 2014), breast cancer (Shu et al., 2016), melanoma (Fontanals-Cirera et al., 2017), and inflammation in activated macrophages (Nicodeme et al., 2010).

In a mechanistic study on how inhibition of the widely expressed transcriptional coactivator BRD4 leads to selective inhibition of the Myc oncogene in multiple myeloma, it was shown that BRD4 and mediator co-occupy in chromatin in a small set of exceptionally large super-enhancers associated with genes important for multiple myeloma. JQ1 led to preferential loss of BRD4 at super-enhancers and transcriptional elongation on those genes, including Myc (Lovén et al., 2013). Super-enhancers are defined as a long class of clusters of regulatory regions bound by TFs that control cell identity and disease genes. Components of super-enhancers also facilitate recruitment of Drosha/DGCR8 for primary-microRNA (pri-miRNA) processing to boost cell-specific miRNA production. Therefore, BRD4 and super-enhancers facilitate transcription of key identity genes, including coding mRNA and biogenesis of master miRNAs that are crucial for cell identity (Suzuki et al., 2017). On the contrary, the testis-specific protein BRDT directs super-enhancer activity in a subset of esophageal squamous cell carcinoma (ESCC). BRDT is aberrantly expressed in over 30% of ESCC, enhances ΔNp63 (a defining factor of the squamous subtype)-dependent SE-associated genes, and controls the migratory potential of ESCC cells (Wang et al., 2021b). The concept of super-enhancer also led to more studies to identify key genes and reveal mechanisms of BET proteins in disease-related cell types and genes such as rheumatoid arthritis synovial fibroblasts (Krishna et al., 2021), ESC pluripotency genes (Di Micco et al., 2014), and senescence-associated secretory genes (Tasdemir et al., 2016).

## Bromodomain and Extra-Terminal Domain Proteins in Condensate Formation

Liquid–liquid phase separation, characterized by protein and other bimolecular condensates, has emerged as a crucial membrane-less compartment implicated in biological processes including chromatin reorganization, as well as in human diseases such as neurodegenerative disorders and cancer (Krainer, 2021) (Figure 4). One major function of BRD4 in organization and regulation of eukaryotic genomes is through co-condensate–mediated concentration of transcriptional apparatus. For example, phase separation of DNA-binding cofactor TEAD4, coactivators BRD4 and MED1, and elongation factor CDK9 locally concentrates transcriptional activators to facilitate the expression of TAZ-target genes and TAZ-mediated growth, development, and tumorigenesis (Lu et al., 2020). A link between super-enhancers (SEs) and co-condensates was illustrated by findings that SE-enriched transcriptional coactivators BRD4 and MED1 form nuclear puncta at SEs that exhibit properties of liquid-like condensates to compartmentalize and concentrate transcription apparatus from nuclear extracts (Sabari, 2018). Intrinsically disordered regions (IDRs) of HAT transcription coactivator p300 interact with TF-transactivation domains (TADs), forming co-condensates that promote p300 transactivation and stabilize TF-p300 assembly, priming the recruitment of coactivator BRD4, and contribute to transcriptional bursting regulation and cooperative gene control (Ma, 2021). However, histone acetylation by p300 also antagonizes chromatin phase separation, while BRD4 helps highly acetylated chromatin to form a new phase-separated state with droplets of distinct physical properties (Gibson et al., 2019). Previous reports have shown that protein condensation is mainly driven by the action of electrostatic and hydrophobic interactions that underlies the phase separation of proteins at or below physiological ionic strength, although it was also recently shown that BRD4 can undergo phase separation at a high salt concentration driven by hydrophobic and non-ionic interactions, and is mechanistically different from the low-salt regime (Krainer, 2021). Collectively, these studies demonstrate the versatile functions of BRD4 in the regulation of co-condensate formation through active stabilization of the co-condensates formed between TFs, coactivators, p300, and acetylated histones to attain super-enhancer functions in gene transcription.

## Bromodomain and Extra-Terminal Domain Proteins in Coordination of Gene Transcription and Splicing

It is becoming increasingly clear that transcriptional elongation is intricately coupled with splicing, transcriptional termination, and genome stability (Jonkers and Lis, 2015) (Figure 4). In castration-resistant prostate cancer (CRPC), BET-BD inhibition affects the regulation of alternative splicing of androgen receptor (AR), leading to reduced AR-V7 expression and abrogating AR signaling and growth of CRPC patient–derived models (Welti et al., 2018). In T-cell acute lymphoblastic leukemia (T-ALL) cancer cells and during thymocyte differentiation *in vivo*, distinct patterns of alternative splicing are associated with BRD4 deletion (Uppal et al., 2019). Specifically, BRD4 directly interacts with splicing machinery (FUS, HnRNPM, HnRNPL, U1-70, and U1-A), suggesting interplay of BRD4 and splicing factors that modulates exon usage (Uppal et al., 2019). Finally, BRD4 also regulates splicing during heat shock, as BRD4 depletion leads to significant splicing inhibition (in particular intron retention) in heat-treated cells, leading to decreased mRNA abundancy of affected transcripts, which is mediated by heat shock factor 1 (HSF1)–BRD4 interaction to recruit BRD4 to nuclear stress bodies (Hussong et al., 2017). Collectively, these studies highlight a crucial role of BRD4 in regulation of alternative splicing. More work is needed to address how BRD4 functions to integrate the processes of chromatin structure, transcription, and splicing to ensure proper regulation of gene expression.

## Bromodomain and Extra-Terminal Domain Proteins in DNA Damage Response and Genome Integrity

In addition to transcriptional control, BRD4 also plays a role in DNA double-strand break (DSB) repair (Figure 4). Genome-wide DNA breaks are associated with enhanced H4 acetylation, BRD4 recruitment, and stable establishment of the DNA repair complex, as well as formation of oncogenic gene rearrangements by a non-homologous end joining (NHEJ) pathway. In clinical tumor samples, BRD4 levels are negatively associated with outcomes after prostate cancer radiation therapy (Li et al., 2018). Similarly, BRD2 promotes DNA repair with a spatially restricted chromatin domain. Tip60/KAT5 generates histone H4 acetylation at DSBs, which recruits BRD2 to protect the acetylated chromatin from being deacetylated by histone deacetylases (HDACs) and allows acetylation to spread along chromatin. This process results in the creation of a spatially restricted domain/boundary in chromatin, likely facilitating the binding of repair protein 53BP1 to DSB sites for repair (Gursoy-Yuzugullu et al., 2017).

BRD2 and BRD4 also promote normal DNA replication origin firing and progression, as they bind and recruit TICRR, a key protein required for initiation of DNA replication, to DNA replication origins in hyperacetylated euchromatin (Sansam et al., 2018). BET proteins also promote genome integrity through regulation of replication stress response signaling. In response to exogenous and endogenous replication stress, BRD4 promotes intra-S-phase replication checkpoint CHK1 activation. Therefore, BRD4 inhibition sensitizes cancer cells to various replication stress-inducing agents (Zhang et al., 2018). In addition, BRD4 maintains spatiotemporal coordination of transcription with replication, thus preventing transcription–replication conflict (TRC) and DNA damage checkpoint signaling in oncogenic cells (Edwards et al., 2020; Lam, 2020). As a result, BET-BD inhibition induces HEXIM1- and RAD51-dependent conflicts between transcription and replication, causing replication stress through a rapid overall increase in RNA synthesis (Bowry et al., 2018). Finally, systematic bromodomain protein screens also identified BRD4-mediated R-loop suppression pathways in genome integrity (Kim et al., 2019). R-loops are intermediate structures of transcription that accumulate when transcriptional elongation is blocked by BRD4 inhibitors. In a subset of cancer cells, BRD4 inhibition results in R-loop accumulation, leading to cell death caused by transcription–replication collisions and DNA double-strand breaks during the S-phase (Lam et al., 2020). These studies suggest targeting BET proteins as a new therapeutic strategy of cancer in that inhibition of BRD4 induces cell death of oncogenic cells through mechanisms such as transcription–replication conflict, R-loop accumulation, and DNA damage.

## Strategies Toward Selective Modulation of Bromodomain and Extra-Terminal Domain Protein Functions

### Distinct Functions of Individual Bromodomain and Extra-Terminal Domain Proteins

Small molecules that target BET bromodomains are being tested for various diseases but do not discern between BET proteins. Studies have highlighted context-specific, distinct, and often opposing functions of BRD2, BRD3, BRD4, and BRDT. For example, distinct roles of BET proteins in ERα enhancer function and gene regulation have been demonstrated in breast cancer cells. While BRD2, BRD3, and BRD4 have partially redundant roles at ERα enhancers and gene transcription, a more unique role of BRD3 in ERα^+^ breast cancer is revealed. BRD3 is recruited to a subset of ER-binding sites (ERBSs) enriched with active enhancer features in clusters likely functioning as super-enhancers and associated with highly estradiol (E2)-responsive genes (Murakami et al., 2019). In triple-negative breast cancer (TNBC), BRD2 positively regulates epithelial-to-mesenchymal transition (EMT), whereas BRD3 and BRD4 functionally repress this program with different transcriptional programs (Andrieu and Denis, 2018). Similarly, BRD4 is required for myogenic differentiation, whereas BRD3 represses the differentiation program (Roberts, 2017). In primary pancreatic stellate cells (PSCs) isolated from human pancreatic ductal adenocarcinoma (PDAC) tumors, BRD4 positively regulates collagen I expression in PSCs, while BRD2 and BRD3 negatively regulate it (Kumar, 2017). Importantly, the distinct functions of BRD4 and BRD2 could coordinate mouse ESC (mESC) control of pluripotency and exit that initiate lineage specification. BRD4 interacts with BRD9 in a bromodomain-dependent fashion, leading to the recruitment of GBAF-GLTSCR1 to form a smaller, non-canonical BAF complex, to coregulate the expression of key regulators of naïve mESC pluripotency (Gatchalian, 2018). BRD2 and BRD4 occupy Nodal gene regulatory elements (NREs), and BRD4 downregulation facilitates exit and drives enhanced BRD2 NRE occupancy to promote differentiative Nodal-Smad2 signaling (Fernandez-Alonso et al., 2017). Finally, the aberrant expression of BRDT in esophageal carcinoma (Wang et al., 2021b), ovarian cancer (Chen, 2020), and renal cell carcinoma (Wan et al., 2020) also contributes to the context-dependent function of BET proteins and is an attractive target for selective inhibition, in particular for different cancer types.

The molecular basis for differences in BET protein function within a given lineage has remained elusive. A recent study has performed an interactome profiling of BRD2, BRD3, BRD4, and BRDT interacting proteins and has gained significant insights into their selective interactions. As expected, the authors confirmed some shared interactors across all BET proteins. Notably, they noted that the p-TEFb and NELF complex is commonly associated with BRDT and BRD4, components of negative transcriptional regulators such as the NURD complex are associated with BRD3 and BRD4, while RNA Pol-II subunits and Mediator subunits are only identified as preferentially associated with BRD4. These data highlight the unique but versatile function of BRD4, suggesting an intricate interplay among BET proteins (Lambert et al., 2019). Finally, BRD2 and BRD3 have a similar domain structure and function to BRD4. An early study using fluorescence resonance energy transfer (FRET) to determine the interaction between cyan fluorescent protein (CFP)-tagged BRD2 and yellow fluorescent protein (YFP)-tagged histones provided important insights into the specificity of recognition of acetylated histones by BRD2. Specifically, BRD2 interacts with histone H4K5/K12 but not K8/K16, and to lesser degree H2B, but not other histones (Kanno et al., 2004). Studies highlighted the overlapping and non-overlapping functions of BRD2/3 and BRD4 in gene transcription. In a defined *in vitro* chromatin transcription system, both BRD2 and BRD3 allowed RNA Pol-II to transcribe through nucleosomes depending on specific histone H4 modifications such as H4K5/12 (LeRoy et al., 2008). Interestingly, apart from acetylated histone, selective BRD2 recruitment has shown to be mediated by a histone variant H2A.Z.2, a driver of malignant melanoma to promote melanoma cell survival (Vardabasso et al., 2015). Similarly, selective interaction of BRD2 with Stat3(K87ac) has also been reported in Th17 cells by our own study (Cheung et al., 2017a). The current knowledge has agreed that BRD2 also plays an important role in cooperating with CTCF/cohesin components to define transcriptional and architectural boundaries, organizing chromatin complex formation, while BRD4 performs its function of transcriptional elongation (Cheung et al., 2017a; Hsu et al., 2017). Collectively, these studies suggest the collaborative nature of BET proteins in directing gene transcription programs in chromatin.

Importantly, using a gene-knockout rescue system in a BRD2^−/−^ erythroblast cell line, a series of mutant and chimeric BET proteins are functionally evaluated (Werner et al., 2020). BET N-terminal halves bearing BDs have a marked difference in protein stability but do not account for specificity in BET protein function. The function specificity is indeed mostly determined by the C-terminal half, as illustrated by a chimeric BET protein that consists of BRD4S-N and BRD2-C and functions similarly to intact BRD2. Part of the function for BRD2-C terminal functional specificity can be attributed to the coiled-coil (CC) domain, which functions in concert with the adjacent ET domain to impart BRD2-like activity onto BRD4S (Werner et al., 2020). These studies have collectively indicated that BET proteins in most cases are distinct in functions, which can be due to the intrinsic difference in C-terminal domains, leading to different interactomes that impart distinct functions in various contexts.

## Transcriptional Repressor Functions of Bromodomain and Extra-Terminal Domain Proteins

BRD4 inhibition leads to gene activation indirectly or directly, in contrast to the widely accepted positive role of BRD4 in gene expression. As an indirect mechanism, TIP60/BRD4 represses activation of endogenous retroviral elements and Irf7-mediated inflammatory response through positively regulating the expression of SUV39H1 and SETDB1 and global histone H3K9me3 in colon cancer (Rajagopalan et al., 2018). On the contrary, genes that are bounded by and repressed directly by BRD4 can also have functional significance. For example, BRD4 binds to the promoters of oxidative phosphorylation (OXPHOS) genes to repress the transcription. Upon BET-BD inhibitor treatment, BRD4 is displaced and OXPHOS gene expression increases which corrects bioenergetic deficiency caused by mitochondrial disease complex I mutations (Barrow et al., 2016). The mechanisms of how BRD4 represses gene transcription directly are currently under investigation. The silencing effect of a short isoform of BRD4S as a corepressor has been demonstrated in HIV-1 transcription, through direct interaction of BRD4S with BRG1, a catalytic unit of BAF with known repressive function in HIV-1 transcription, via its bromodomain and ET domain (Conrad et al., 2017). BRD4 can also interact with the methyltransferase G9a to repress a transcriptional program by di-methylating H3K9 that promotes genes involved in autophagy and lysosome biogenesis. BRD4 inhibition or, in the case of starvation of cells, repressive function of BRD4 is relieved, leading to autophagy gene activation and cell survival (Sakamaki et al., 2017; Sakamaki and Ryan, 2017). However, the interaction of BRD4/G9a can only partly explain the repression effect, underlying the need to further identify and characterize BRD4-repressive complexes. Interestingly, RING1B, a subunit of PRC1, colocalizes enhancers containing ERα in ER^+^ cells and to BRD4 enhancers in TNBC cells in active genes. Physical interaction of PRC1 and Fs(1)h and Br140, the *Drosophila* orthologs of BRD4 and BRD1, has also been confimed (Kang et al., 2017; Chan, 2018), indicating the BRD4–PRC1 repressive complex can play a functional role in some contexts yet to be determined. The transcriptionally repressive function of BRDT has also been demonstrated during spermatogenesis. BRDT undergoes complexation with HDAC1, PRMT5, and TRIM28 to transcriptionally repress the testis-specific histone *H1t* gene (Wang and Wolgemuth, 2016). Taken together, while previous studies demonstrated the dominant role of BET proteins in transcriptional activation, these studies have added another layer of complexity by showing that the context-dependent BET-interacting repressive complex can repress gene transcription with functional significance. Therefore, it will be necessary to evaluate the therapeutic efficacies of strategies selectively targeting the BET-repressive complex in the future.

## BRD4 Short Isoform *vs* Long Isoform

The expression levels of BRD isoforms BRD4L and BRD4S seem to be determined by the function of splicing kinase SRPK1, which influences BRD4 occupancy at genomic loci of myc and bcl2 in leukemogenesis (Tzelepis, 2018). There are currently three identified BRD4 isoforms, and their functional distinctions are beginning to be unraveled, pointing to the opposing role of BRD4S *vs.* BRD4L forms. In human cancer cells, BRD4S forms nuclear puncta that possess liquid-like properties and colocalizes with BRD4L, MED1, and histone H3K27Ac. BRD4 puncta correlate with BRD4S but not the BRD4L expression level. BRD4S plays a substantially larger role than BRD4L in incorporating BRD4 condensations in chromatin and is facilitated by its tandem BDs binding to lysine-acetylated histones (Han et al., 2020). Function of BRD4S has been demonstrated in the context of human papillomaviruses (HPVs) that encode E2 protein to recruit viral and cellular proteins. BRD4 is a highly conserved interactor with E2 protein. BRD4S lacks the CTM but has an intact phosphor-dependent interaction domain (PDID) and basic interaction domain (BID) and can interact with HPV31 E2 through the PDID and repress E2 activities, in contrast to the activator function of BRD4L on E2-mediated viral gene transcription (Yigitliler, 2021). Opposing functions of BRD4 isoforms have also been studied in breast cancer, showing the oncogenic EN1–BRD4S axis and tumor-suppressive BRD4L in breast cancer, which demonstrates the context-dependent functions of long and short isoforms (Wu et al., 2020). As aforementioned, the function of BET proteins is mostly determined by the C-terminal half and the associated interactome that dictates their functions in specificity contexts; it is therefore important to characterize the functional distinctions of BRD4L *vs.* BRD4S in different disease contexts.

## Toxicity of Bromodomain and Extra-Terminal Domain Protein Inhibition *In Vivo*

BET-BD inhibitors have shown therapeutic efficacies in preclinical models. However, the role of BRD4 in the normal cell developmental process and subsequently sustained BET-BD inhibition in normal tissue toxicity has been reported in clinical evaluation of various BET-BD inhibitors but not well characterized. Analysis of Brd4-KO mice using the Brd4^fl/fl^ system has shown that BRD4 is required for hematopoietic stem cell expansion and progenitor development but has a limited role in macrophage development (Dey, 2019). On the contrary, using an inducible and reversible transgenic RNAi mouse model, Lowe and colleagues showed that sustained suppression of BRD4 in adult mice displayed reversible epidermal hyperplasia, alopecia, and decreased cellular diversity and stem cell depletion in the small intestine (Bolden et al., 2014). These studies therefore highlight the ubiquitous functions of BRD4 in different tissues. They also suggest that although BET-BD inhibition can be developed as a therapeutic approach for inflammatory diseases due to the reversibility of toxicity, treatment protocols need to be optimized to achieve therapeutic efficacy while avoiding prolonged suppression to allow for tissue recovery.

## Selective Function and Targeting of Bromodomain and Extra-Terminal Domain BD1 *vs.* BD2 in Drug Development

To minimize the toxicity of pan-BET protein inhibition *in vivo*, recent studies have focused on developing strategies that selectively target individual BET proteins or individual bromodomains. Importantly, this strategy has also been demonstrated with beneficial effects in cancer treatment. BET-BD1 and BET-BD2 selective inhibitors have been shown to be better therapeutic strategies for cancer or inflammatory diseases, respectively (Gilan et al., 2020), as toxicity associated with single BET-BD inhibition appears to be significantly lower than that of pan-BET-BD inhibition (Faivre et al., 2020). However, the dichotomous usage of BET-BD1 and -BD2 inhibitors in cancer and inflammatory diseases needs to be further refined with more investigation of their detailed mechanism of action. Cheung *et al.* reported that BRD4-BD1 inhibition selectively blocks T-helper (Th)17 cell differentiation and ameliorates colitis (Cheung et al., 2017b). Our unpublished results also have revealed that selective targeting of BRD4-BD2 inhibits Th2 cell differentiation, thereby paving the way for the applications of BRD4 BD1- *vs.* BD2-selective inhibitors to selectively target Th17- or Th2-related immune diseases.

## Conclusion

The major advances in bromodomain biology in the recent years have greatly enhanced our mechanistic understanding of the diverse and versatile functions of BET proteins in the complex regulation of gene transcription in chromatin in biology as well as human diseases. It has become increasingly evident that BET proteins exert distinct and overlapping functions collectively with many other key transcription and chromatin regulatory proteins that enable cells to execute gene transcription programs in response to physiological and pathophysiological cues. It is therefore necessary to characterize and understand the context-specific functions of BET proteins for the purpose of developing novel small molecule bromodomain inhibitors that are capable of selectively modulating BET protein functions to control gene transcription in a given functional setting. Such bromodomain inhibitors likely have great potential as new epigenetic therapies for a wide array of human diseases including cancer, inflammatory and autoimmune diseases, and cardiovascular disorders.
